# The Comparison of a New Ultrasound-Induced Depression Model to the Chronic Mild Stress Paradigm

**DOI:** 10.3389/fnbeh.2019.00146

**Published:** 2019-07-02

**Authors:** Yana A. Zorkina, Eugene A. Zubkov, Anna Yu. Morozova, Valeriya M. Ushakova, Vladimir P. Chekhonin

**Affiliations:** ^1^Department of Basic and Applied Neurobiology, V.P. Serbsky National Medical Research Center for Psychiatry and Narcology, Moscow, Russia; ^2^Department of Medical Nanobiotechnology, Pirogov Russian National Research Medical University, Moscow, Russia

**Keywords:** depression, stress, rats, ultrasonic, chronic mild stress, animal model

## Abstract

Willner’s “chronic mild stress” (CMS) model is a globally recognized and most commonly used depression model. A depression model induced by ultrasonic exposure of variable frequencies has been created in our laboratory. This article compares two models of the depressive-like state according to three validity criteria. *Face validity* has been demonstrated in sucrose preference test, Porsolt test, social interest, open field and the Morris water maze. Rats after ultrasound impact have more pronounced anhedonia and social isolation. *The construct validity* has been proven due to increased levels of corticosterone, epinephrine and norepinephrine and reduced levels of dopamine and some of its metabolites in rat plasma after ultrasound exposure. *Predictive validity* has been described previously, where the therapeutic effects of various classes of antidepressants have been shown. Our study has demonstrated that the ultrasound-induced depression model is suitable, such as the generally accepted CMS protocol, and meets all required validity criteria. The model presented in this article might help to study pathogenetic mechanisms of depressive disorders, as well as to test promising methods of depression treatment.

## Introduction

At a global level, over 4.4% of the world’s population is estimated to suffer from depression (WHO, 2017). The creation of models in psychiatry has its limitations due to the exceptional specificity of the human psychology including the presence of highly differentiated structures of the neocortex that form a functional unit responsible for consciousness and thinking, as well as for speech as a second signal system (Vocate, 1987). Depressions are divided into reactive (stress-induced) and endogenous conditionally induced (Malki et al., 2014). Stress-induced models include: deprivation models, models created by pain shock, placement in extreme conditions, immobilization, forced swimming, social isolation, and mice social defeat model. Sixty-six percentage of all articles published during the year 2018 about stress-induced depression are based on Willner’s protocol. In this model, rats are exposed to stressors that change unpredictably resulting in depressive-like conditions in animals (Willner et al., 1987). The recently published meta-analysis shows that chronic mild stress (CMS) protocol is strongly associated with anhedonic behavior in rodents. The authors point out the heterogeneity in the animals’ responses, even in individual subgroups, because of the many variations of this protocol (Antoniuk et al., 2019). However, there is still no adequate and well-reproducible model describing a depressive-like state corresponding to that in humans.

The listed above protocols do not cause psychological trauma, without physical impact on the animal. Social isolation or separation from the mother may be partially considered models of psychological stress. But it is impossible to extrapolate these results to humans, because early social isolation does not always lead to depression in adulthood, and in adults, it causes other mental disorders (Tan et al., 2017).

Models of psychological trauma in animals are few; besides, protocols used to create such models contain, for the most part, stress stimuli that differ greatly from the negative factors affecting humans in the society in developed countries, namely, they do not focus on the chronic informational uncertainty. Therefore, there are no available animal models of depression-like behavior, where informational uncertainty, concerning for example terrorist acts, wars, catastrophes, as well as economic and social instability uncertainty would be the stress factor, being the main factor causing depression in humans (Neumann et al., 2011).

Informational uncertainty in rodents with subsequent induction of depressive-like behavior can be caused by exposure to ultrasound (US) of variable frequency. Exposure to US with a frequency of 20 kHz to 45 kHz simulates the information flow carrying a negative emotional load (Takahashi et al., 2011) and forms a state of learned helplessness, which is interpreted as a depressive-like state in animals. The first article about the new depression model was published in 2013 (Morozova et al., 2013a). At the beginning of the development of our US paradigm, we conducted the study that evaluated the impact of “white noise” on rats’ behavior. According to previous work (Morozova et al., 2016), a comparison of a 3-week exposure to 50 dB-ultrasound of mixed frequencies (“white noise”) at the range of 16–20 kHz did not result in a depressive-like state. The exposure of single frequency US-wave, 22 kHz, leads to anxiety in the first 5 min after exposure (Demaestri et al., 2019), but continuous action of 22 kHz does not lead to behavioral changes, because of an adaptation to the unchangeable stress impact (da Oliveira et al., 2015).

The aim of this article is to compare the CMS model with the ultrasound-induced depression model which has been developed in our laboratory.

## Materials and Methods

### Animals

Experiments were performed on male Wistar rats (*n* = 60) that were 2.5 months old. The animals were provided by Pushchino, RAS, Moscow region. Experimental animals (US and CMS group) were housed individually in polycarbonate transparent cages (42 × 26 × 15 cm) during stress protocols. Control group were housed in groups of five animals in polycarbonate cages (55 × 35 × 20 cm). After the stress protocol rats from CMS and US group were placed in cages in groups. There was no group of experimental animals which was housed individually because our previous study showed that 3-week isolation does not lead to depressive-like behavior (Gorlova et al., 2018). They were maintained on a 12-h light/dark cycle under controllable laboratory conditions; food and water were available *ad libitum*. Housing conditions and all experimental procedures were set up and maintained in accordance with Directive 2010/63/EU of 22 September 2010 and approved by the local ethical committee of V.P. Serbsky National Medical Research Center for Psychiatry and Narcology.

### Study Design

The animals were divided into the following experimental groups: control animals (*n* = 20); rats exposed to ultrasonic radiation for 3 weeks (*n* = 20); and rats exposed to CMS protocol (*n* = 20). On the next day after CMS and US exposure, the sucrose preference test was performed. The tests were conducted with a delay of 1 day between them in the following order: social interest test, open field, forced swim and Morris water maze.

### Ultrasonic Exposure

**Figure 1:**
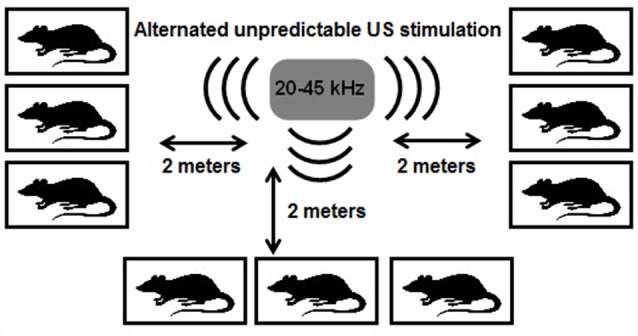
Scheme of ultrasound (US) impact. All rats were placed in individual cages in equal distance (2 m) from US generator. It generates US waves of variable frequencies from 20 to 45 kHz. US exposure was performed 24-h per day during 3 weeks.

The US exposure was performed for 24 h each day during 3 weeks and consists of periods between following range: low frequencies (20–25 kHz), middle range frequencies (25 < × < 40 kHz) and frequencies of high range (40–45 kHz). The ultrasound frequencies changed every 10 min. Low and middle frequency ultrasound constituted 35% of emission each, high frequencies constituted 30% of emission time. The loudness of the sound was fluctuating at the range ±10% of the averaged value, i.e., 50 ± 5 dB. Rats communicate with intensity up to 86 dB, so 50 dB is normal volume of sound (Smith, 1979). The ultrasonic device (Weitech, Belgium) was suspended from the ceiling, and the loudspeaker was oriented downwards, where there were cages with rats at a distance of 2 m (Figure 1). The position of the cages was changed every 3 days. The rats exposed to US were kept in the separate room in equal conditions with control and CMS groups.

### CMS Protocol

The CMS was performed for 3 weeks during following protocol: Monday 10 a.m.–6 p.m. food deprivation, Monday 6 p.m.–Tuesday 10 a.m. intermittent lighting (off/on every 2 h), Tuesday 10 a.m.–6 p.m. 45° cage tilt, Tuesday 6 p.m.–Wednesday 10 a.m. water deprivation, Wednesday 10 a.m.–6 p.m. stroboscopic illumination in the dark, Wednesday 6 p.m.–Thursday 10 a.m. light in the night; Thursday 10 a.m.–6 p.m. Food deprivation in soiled cage (water in sawdust; 250 ml of water in each cage), Thursday 6 p.m.–Friday 10 a.m. mouse cage, Friday 10 a.m.–6 p.m. intermittent lighting (off/on every 2 h), Friday 6 p.m.–Sunday 10 a.m. paired housing (new partner 6 month old), Sunday 10 a.m.–6 p.m. stroboscopic illumination in the dark, Sunday 6 p.m.–Monday 10 a.m. light in the night.

### Behavior Tests

#### Social Interest Test

Social interaction test was performed as described previously (Morozova et al., 2016). The duration of social interaction was defined as any pro-active contacts that the rat has shown towards the juvenile male, which typically comprised of approaches, body contacts, following, sniffing and exploration, and was scored using RealTimer software (OpenScience, Russia).

#### Sucrose Test

During this test, rats were given a free choice between two bottles for 24 h, one with a 1% sucrose solution and another with tap water (Morozova et al., 2016). No previous food or water deprivation was applied before the test.

#### Open Field Test

The open field test was carried out in a round arena (d = 120 cm, h = 50 cm) made from gray plastic and illuminated with white light (25 lx). The animal was placed near the wall and its movements were tracked for a 5 min period with a digital camera which was placed above the arena. The number of crossed squares (horizontal locomotor activity) and the number of rearings (vertical activity) were evaluated using RealTimer software (OpenScience, Russia).

#### Forced Swim Test

A transparent cylindrical pool made of glass (diameter 15 cm, height 40 cm) was filled with water (24°C) to a level that prevented a rat from touching the bottom. Animals were put in the pool for 8 min. The absence of any directed movements of an animal’s head and body was considered as immobility and was kept track of during the last 6 min of the test. We used the following protocol with some modification (Bourin et al., 2004).

#### Morris Test

Morris water maze was performed following standard procedures (Morris, 1984). The Morris water maze was represented by a round pool (diameter 150 cm) with gray walls. The pool was filled up with clear water (depth 40 cm, water temperature 24°C). The round plastic transparent platform (diameter 8 cm) was placed to the center of one of the pool quadrants and 2 cm under the surface. Training consisted of eight sessions. Each animal was placed into the water in different places. Swimming time was recorded until the animal reached the platform. Rats that were unable to find the platform for 60 s were then directed to the platform by a hand. Upon reaching the platform, the rats were left on it for 15 s. The interval between training sessions was 1 min. 48 h after training, the latency to find the platform was measured in one 60 s session. The orienting points were located around the maze on the walls.

### Plasma Corticosterone and Catecholamine Levels

The plasma was collected from tail vein at the last day of US exposure at 11 a.m. in the tubes with heparin at the end of ultrasound exposure. The plasma corticosterone level was determined using a commercial ELISA assay kit (Cat. No: AC-14F1) supplied by Immunodiagnostic Systems Holdings PLC, United Kingdom according to the manufacturer’s protocol.

Plasma catecholamine level was performed using HPLC. A chromatographic system with a pump PM-80 (BAS, USA) and an LC-4B electrochemical detector (BAS, USA) was used. Analytical column: Hypersil BDS-C18, 3 μm, 4.0 × 100 mm (Phenomenex, USA).

### Statistical Analysis

Analysis was performed using STATICTICA software version 10.0. All groups in all tests were tested for normal distribution by Shapiro-Wilk test. The data in Social interest, Sucrose test, Open field (squares and rearings), FST, level of corticosterone, catecholamine and its metabolites have normal distribution (data are expressed as Mean ± SD). Then one-way ANOVA followed by Fisher’s *post hoc* LSD test was applied to compare these groups. Kruskal-Wallis test with Newman-Keuls *post hoc* test was used to compare groups in Morris test, the data in this test did not have normal distribution (data are expressed as median ± quartile). The alpha level was set as *p* < 0.05.

## Results

The plasma level of corticosterone, epinephrine, norepinephrine was increased twice as high compared to control. The level of dopamine and its metabolites were decreased (see Table 1). Overall we observed the disturbance in catecholamine’s concentration in blood that agrees with the conception of developed depressive-like behavior.

**Table 1:** Level of corticosterone, catecholamine and it metabolites (nmol/l).

	Epinephrine	Norepinephrine	Dopamine	DOPA	DOPAC	Corticosterone
Control	3.03 ± 1.79	2.3 ± 1.26	11.17 ± 2.58	8.83 ± 3.17	10.27 ± 2.3	302.24 ± 144.15
US	5.78 ± 1.9**	7.6 ± 1.16**	5.77 ± 2.4**	4.05 ± 1.78**	5.38 ± 1.98**	602.65 ± 115.35**

All substances were measured by HPLS, except for corticosterone (ELISA). Data presented as Mean ± SD. **p < 0.01 compared to control. *F*_(1,38)_ = 8.75; 82.5; 12.9; 14.15; 23.3; 15.89, respectively.

**Figure 2:**
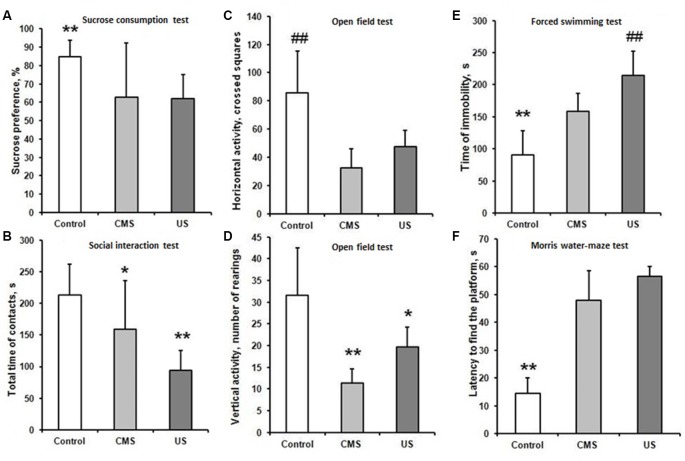
Behavior tests. CMS—rats exposed to chronic mild stress, US—rats after 21-days ultrasound exposure. **(A)** Sucrose preference test. Preference index was calculated according to the formula: (V_s_+ V_w_)*100%, where V_w_ is volume of consumed water, V_s_ is volume of consumed sweet water. Data presented as Mean ± SD. Data compared using one-way ANOVA with *post hoc* Fisher’s LSD test. The sucrose preference index in CMS and US groups did not differ from each other and was significantly lower compared to control (**p < 0.01 compared to US and CMS groups). **(B)** Social interest test. Time of social contacts between the experimental animal and the juvenile male. Data presented as Mean ± SD. Data compared using one-way ANOVA with *post hoc* Fisher’s LSD test. Time of social contacts was reduced in CMS group compared to control (*p < 0.05). In US group it was decreased more significantly (**p < 0.01 compared to control and CMS). **(C,D)** Open field test. Comparison of horizontal activity (number of crossed squares, **C**) and vertical activity (number of rearings, **D**) between control, US and to CMS groups. Data presented as Mean ± SD. Data compared using one-way ANOVA with *post hoc* Fisher’s LSD test. The number of crossed squares was less in CMS and US group compared to control (^##^*p* < 0.01). The experimental groups were not differing from each other. The number of rearings was less in CMS and US group compared to control (**p < 0.01 and *p < 0.05, respectively). **(E)** Forced swimming test (Porsolt test). Comparison of immobility time during the last 6 min of an 8 min swim session between control, US and CMS groups. Data presented as Mean ± SD. Data compared using one-way ANOVA with* post hoc* Fisher’s LSD test. Time of immobility was increased in CMS and US group compared to control (**p < 0.01). In US group it was increased more significantly (^##^*p* < 0.01 compared to CMS). **(F)** Morris water maze test. Test session 48 h after learning. Data presented as Median ± quartile. Data compared using one-way ANOVA with* post hoc* Fisher’s LSD test. Time finding the platform was increased in CMS and US group, stressed groups did not differ from each other (**p < 0.01).

A decrease in sucrose preference index in CMS (62.7 ± 29) and US (62 ± 13%) groups compared to control (85 ± 8.7%; *F*_(2,57)_ = 11.57; *p* < 0.01) revealed the development of anhedonia. Although no statistically significant difference was found between the CMS and US groups, SDs in CMS group were bigger (Figure 2A). The large SD in CMS group can be explained because 30% of animals did not show anhedonia. Total liquid intake did not differ between all experimental groups (*F*_(2,57)_ = 0.124; *p* = 0.88).

Impact of US led to a marked reduction of social activity in the social interaction test in the US group (94.6 ± 30.5) and CMS group (159.1 ± 72.6), compared to the control group (213.5 ± 54.3; *F*_(2,57)_ = 17.86; *p* < 0.01; Figure 2B). Social interaction in the CMS group was higher than the control group (*p* = 0.04). Contrary to that, the US group has much more pronounced differences to the control group (*p* < 0.01). Experimental animals after US exposure avoided contact with the juvenile male in every way, and in some cases showed freezing behavior when infant rat came into contact with them.

The number of squares and rearings in open field test has significant difference between experimental groups and control (*F*_(2,57)_ = 17.16 and *F*_(2,57)_ = 17.26 accordantly, *p* < 0.01). There was no difference between CMS and US group (Figures 2C,D). Thus, the stressed rats expressed marked decrease in exploration behavior.

In the forced swimming test US and CMS groups showed a significantly longer immobility time: US (215 ± 37.7) and CMS (159 ± 27.8) compared to the control group (91 ± 36.7; *F*_(2,57)_ = 79.42; *p* < 0.01). The time of immobility was the highest in US group (*p* < 0.01 compared to CMS and control), the rats after CMS were less immobile than rats after US exposure (*p* < 0.01 CMS compared to control; Figure 2E). Although all experimental groups showed despair behavior, rats after US exposure struggled less than those in the CMS group.

In the Morris water maze the time of finding platform had significant difference from control–14.5 (7.8;5.5; *H*_(2,60)_ = 12.84; *p* < 0.01 for CMS and US group), but did not differ between the CMS–48 (13;10.5) and US–56.5 (25.3;3.5) group (Figure 2F). Therefore, mild stress altered long term spatial memory in the same manner as ultrasound protocol.

## Discussion

As it was emphasized by Willner, “Animal models of psychiatric states may be defined as procedures applied to laboratory animals that engender behavioral changes, which are intended to be homologous to aspects of psychiatric disorders, and can, therefore, be used as experimental tools to further the understanding of human psychopathology” (Willner, 2013). Most animal models of depression are based on the induction of a depressive-like behavior by stress. Among them, the CMS model is the most widely tested one (Antoniuk et al., 2019).

To evaluate the model as the one that is appropriate and adequately reproduces mental disorders, Willner proposed the following validity criteria: face validity, predictive validity, construct validity.

### Face Validity

This criterion implies compliance of the model based on its behavioral manifestations. Sucrose preference, social interest and forced swim test are used to determine the depressive-like state in animals since there is a decrease in social interaction and sings of anhedonia in depression in both animals and human. In our study, in both models, the level of sucrose preference decreased and did not differ in the experimental groups. However, in 30% of rats subjected to CMS, the sucrose preference index was more than 90%, as in reference group animals, which suggests that rats after CMS consumed the same amount of sweet water as healthy animals. At the same time, in the ultrasound-induced depression model, such findings were not observed. The maximum preference index in US group was 75%. The fact that many of the CMS rats did not reduce sucrose intake in the sucrose preference test is actually not that surprising. On this basis, some researchers divide rats into susceptible and resistant to stress effects (Wiborg, 2013). Apparently, US exposure makes the threatened group more homogeneous and has a stronger effect on animals, although has no physical impact.

The forced swim test is used for the evaluation of depression (Kraeuter et al., 2019). In this test, rats after ultrasound exposure showed significantly higher immobility time than rats after CMS, about twice as much as in the control group.

In the social interest test, the group of animals after the CMS showed the decrease in time of social contact, but the difference between US group and control was much more remarkable, compared to the difference between CMS and control group. It is related to the fact that 40% of animals showed no decrease in social activity below 200 s; and three of these animals did not demonstrate anhedonia, either. The US group showed greater homogeneity with a lower standard deviation; the average of social contacts did not exceed 150 s. Therefore, according to three tests demonstrating a depressive-like state we found no signs of anhedonia and reduced social interaction in six animals; although the immobility time of these animals has been raised and did not exceed the minimum time of immobility in the US group. Therefore, if we take into account the development of depressive-like state by behavioral parameters of rats in all experimental tests, we can conclude that 30% of animals with the CMS model have not shown depressive-like state simultaneously in all three tests. Not only results of this study, but also already published data confirm this (Willner, 2017b). Immobility time, sucrose preference index, time of social contacts in US-induced depression model gave similar results in all experimental series. CMS protocol is often criticized for the non-reproducibility of behavioral test results. Willner himself confirms it having analyzed hundreds of publications made based on his model (Willner, 2017b). The meta-analysis of Willner’s model also indicated this fact, except for anhedonia (Antoniuk et al., 2019). It should be noted that the test results of the failed CMS model are not likely to be published. However, we were able to find articles that discuss the failed test of this model (Krupina et al., 2012). The low reproducibility is primarily due to the ambiguity of the protocol used. Willner’s model has been known since 1987. There are 230 publications from 180 labs in more than 30 countries (Willner, 2017a), and in the majority of these publications, the protocols are different. Even if types of stress stimuli are the same as in original Willner’s articles, they do not have the same order, and the duration of CMS also differs. The variability of stressors and the human factor also contribute to the ambiguity: the variability of new partners (weight and age) and the ambiguity of the term “small and wet cage.”

Willner’s model involves the impact on the senses of animals (light) or physical discomfort (placement in wet sawdust, small cage), reversal of the day/night cycle, deprivation of water and food, and a new partner. Only some of these stress factors are present in the daily life of a human. As Willner says, animal models of psychiatric conditions can be defined as procedures applied to laboratory animals that produce behavioral changes; these are designed to be homologous to aspects of mental disorders and can, therefore, be used as experimental tools for further understanding human psychopathology (Willner, 2013). Physical discomfort and stress associated with the lack of water and food is not the main type of stress in developed countries, where depression is diagnosed with an incidence of up to 45% (Roohafza et al., 2011). Therefore, chronic information stress may be the stress which is similar to humans; in our animal experiments, it is caused by exposure to ultrasound of variable frequency. Therefore the US-induced depression model has some advantages due to the type of stress stimulus which is closer to the aspects of real-life situation observed in the human population.

Depression is also often accompanied by cognitive and motivational disorders. Long-term spatial memory, learning ability, locomotor activity in open field test and Morris water maze were also tested after two stress protocols. The search time for the platform in the Morris water maze in CMS and US experimental groups of animals exposed to different types of stress did not differ, but it was longer than that in the control group. This suggests impairment of long-term spatial memory in rats, which is consistent with symptoms that occur in depression. According to this aspect of validity, both depression modeling protocols are equally good.

In the open field test, the parameters of locomotor and exploratory activities were investigated. The number of crossed squares and rearing did not differ in the experimental groups; it was significantly lower in CMS and US group compared to control.

### The Construct Validity

This criterion suggests that the modeled depression features should be unambiguously interpreted and regarded as homologous, and should be related to depression both empirically and theoretically. The most powerful trigger for the development of depression is stress, which causes the depression symptoms. Under normal conditions, the response to stress is an integral part of adaptation. However, repeated stress affects the adaptation system. This potentiates the central nervous system vulnerability to depression and other disorders (McIsaac and Young, 2009). It is known that negative psychological factors lead to activate hypothalamic-adrenal axis in human (Dallé and Mabandla, 2018). Stress in rats leads to an increase in the plasma level of glucocorticoids, such as corticosterone, and catecholamine, such as epinephrine and norepinephrine (Smith and Vale, 2017). CMS also increases the normal steroid concentration in experimental animals (Azpiroz et al., 1999). Besides, basic and clinical studies demonstrate deficits and dysregulation of the dopaminergic system in depression (Belujon and Grace, 2017). In CMS models of depression, the normal dopamine concentration in blood serum and brain tissue is disturbed (Bekris et al., 2005). According to our data, the concentration of corticosterone, epinephrine and norepinephrine in the blood increased twice as high on the 21st day of ultrasound exposure, at the same time the concentration of dopamine and its metabolites was decreased. According to literature, anhedonia is induced by dopamine system dysregulation. This dysregulation is caused by failure of stress compensatory mechanisms (Belujon and Grace, 2017). Consequently, our US protocol meets the criteria of construct validity.

### Predictive Validity

For the model of depressive-like behavior in animals, it primarily means a response to antidepressant therapy. Several classes of antidepressants were tested in our US-induced depression model: fluoxetine (a selective serotonin reuptake inhibitor), tianeptine (monoamine reuptake modulator), bupropion (a selective norepinephrine–dopamine reuptake inhibitor), which restored the behavioral test parameters to the level of untreated animals (Table 2; Morozova et al., 2013b). Moreover, the antidepressant effect of electroconvulsive therapy has also been demonstrated in our laboratory (Morozova et al., 2015), without negative effects on memory (Ushakova et al., 2017). According to the predictive validity, the US-induced depression model has all the necessary characteristics. Extensive use of the Willner’s model for testing different types of antidepressants shows the predictive validity in this model, too.

**Table 2:** Antidepressant’s action in ultrasound-induced depression (US) model.

	Social interaction test	Porsolt test	Sucrose preference test
Control	216 ± 50 s**	83 ± 19 s^##^	78 ± 6%^$$^
US	107 ± 33 s	211 ± 48 s	56 ± 15%
US + Fluoxetine	228 ± 41 s	128 ± 56 s	69 ± 10%
US + Bupropion	143 ± 30 s	70 ± 16 s	78 ± 21%
US + Tianeptine	236 ± 40 s	106 ± 24 s	71 ± 8%

Antidepressants were given during 21-days of US protocol. Data presented as Mean ± SD. In social interaction test, we evaluated time of contacts between experimental animal and juvenile rat (s). Time of social contacts was decreased in US group and corrected after antidepressant’s treatment, except for bupropion. **p < 0.01 compared to US group and US + Bupropion. In Porsolt (forced swim) test, we compared immobility time (s). Time of immobility was increased in US group and corrected after antidepressant’s treatment. ^##^*p* < 0.01 compared to US group. In sucrose preference test index was calculated according to the formula: (V_s_ + V_w_)*100%, where V_w_ is volume of consumed water, V_s_ is volume of consumed sweet water. The index was decreased in US group and corrected after antidepressant’s treatment. ^$$^*p* < 0.01 compared to all groups. Data included in the table was taken from our previous study (Morozova et al., 2013b) to emphasize the predictive validity of US-induced depression model.

In Willner’s model, depressive-like behavior persists for up to 3 months (Willner, 1997) and at least 2 months in our model (Morozova et al., 2016).

It should be noted that labor usage, a parameter that is also very important in the reproduction and modeling of psychopathology, is very low in the US-induced depression model. At the same time, Willner’s model implies daily procedures to be performed with the animal by the experimenter.

The ultrasonic model of depression is better than the CMS model from the point of view of animal’s injury and ethics (Nuno, 2013).

Although the Willner’s model has some drawbacks, it is most widely used in the world, therefore, using this model, researchers approached the understanding of the molecular mechanisms of depression and the study of its therapy.

In this study, modeling of the depressive-like state in animals was performed using two different protocols: Willner’s model, the most commonly used model of depression and a new model developed in our laboratory, which involves a 3-week exposure to chronic information stress caused by exposure to US of variable frequency. This ultrasonic exposure is considered a chronic stress due to the fact that these signals carry a negative information load, to which the animal cannot adapt. In both models, there are cognitive symptoms and locomotor and exploratory activity impairment which are also typical for the depressive-like state; and they are equivalent in both depression models. According to the face validity criteria, the US-induced depression model in some tests shows more pronounced depressive-like behavior over Willner’s protocol; and according to the construct and predictive validity criterion, these models are similar. However, we believe that the uniqueness of the protocol applied and the large homology of the type of stressor used in the US-induced depression model are its main advantages. Therefore, we can conclude that the model developed in our laboratory is not only equal to the most frequently used CMS model, but it is better in some aspects such as labor usage, ethical restrictions, easy and uniform protocol without physical impact. We hope the global scientific community will make use of the US-induced depression model in the future to obtain further data to study pathogenetic mechanisms of the depressive-like disorders caused by stressors, which are the closest ones to those present in everyday human life, as well as to test promising depression treatment options.

## Data Availability

All datasets generated for this study are included in the manuscript.

## Ethics Statement

Housing conditions and all experimental procedures were set up and maintained in accordance with Directive 2010/63/EU of 22 September 2010 and carried out under approval of the local veterinary committee.

## Author Contributions

YZ, EZ and AM planned, carried out the experiments and wrote the manuscript. VU took part in the experiments and VC revised the manuscript.

## Conflict of Interest Statement

The authors declare that the research was conducted in the absence of any commercial or financial relationships that could be construed as a potential conflict of interest.
